# TOX‐AD: Imaging of Environmental Neurotoxicants Accumulated in Mouse Brains

**DOI:** 10.1002/alz70855_101024

**Published:** 2025-12-23

**Authors:** Olga Minaeva, Kevin P Kotredes, Juliet A Moncaster, Ning Hua, Zeynep Sarica, Nicola Lupoli, Paul R Territo, Gareth R Howell, Lee E Goldstein

**Affiliations:** ^1^ Boston University Chobanian & Avedisian School of Medicine, Boston, MA, USA; ^2^ The Jackson Laboratory, Bar Harbor, ME, USA; ^3^ Indiana University, Indianapolis, IN, USA; ^4^ Stark Neurosciences Research Institute, Indiana University School of Medicine, Indianapolis, IN, USA; ^5^ Boston University Alzheimer's Disease Research Center, Boston, MA, USA

## Abstract

**Background:**

Age, genetics, and environmental exposure are among the primary risk factors for Alzheimer's Disease (AD) and related dementias (ADRDs) [1]. Exposure to environmental toxicants is disproportionately high in disadvantaged groups, making research on this topic both a medical and social justice concern. This study (**TOX‐AD: 1U01AG088683)** focuses on exposure to lead (Pb), cadmium (Cd), and arsenic (As), each listed among the WHO “Top 10” chemical toxicants of public health concern [2]. We hypothesize that these neurotoxicants alter the expression of AD‐linked genes and potentiate AD pathobiology in a toxicant‐specific, neurodevelopment‐sensitive, biomarker‐responsive, and age‐dependent manner.

In this project, we used laser ablation inductively coupled plasma mass spectrometry (LA‐ICP‐MS) imaging to conduct multi‐elemental brain mapping with ultra‐trace elemental quantification and precise anatomical localization. We employed LA‐ICP‐MS imaging to investigate neurotoxicants' accumulation and regional distribution in mouse brains following chronic exposure to Pb, Cd, and As in drinking water.

**Method:**

The study utilized MODEL‐AD mice of both sexes to establish levels of metal/metalloid retention in the brain and blood after 30‐day exposure to Pb (200 ppm), Cd (5 and 50 ppm), As (20 ppm), control (no added metal) in drinking water. Brains and blood were collected, frozen using liquid nitrogen, and stored at ‐80C. Tissue concentrations of exogenously administered neurotoxicants (Pb, Cd, As) and endogenous biometals (Zn, Cu, Fe) were evaluated by inductively coupled plasma mass spectrometry (ICP‐MS) solution analysis and LA‐ICP‐MS imaging.

**Result:**

ICP‐MS results showed robust Pb, Cd, and As uptake in mouse brain and blood after 30‐day exposure to epidemiologically relevant levels of each metal/metalloid in drinking water. We also demonstrated a strong correlation between brain and blood levels of As, Cd, and Pb. LA‐ICP‐MS mapping showed that neurotoxicants have non‐homogeneous element‐specific accumulation in different brain regions and subregions, including the hippocampus and cortex.

**Conclusion:**

MODEL‐AD mice exposed to Pb, Cd, or As accumulate neurotoxicants in blood and brain that can be detected, imaged, and quantified by ICP‐MS solution analysis and LA‐ICP‐MS imaging.

**References**:

[1] R.A. Armstrong, Risk factors for Alzheimer's disease. *Folia Neuropathologica*., **2019**, *57*, 87–105.

[2] World Health Organization. 10 chemicals of public health concern. **2020**.

